# Supplementary material for: Digital Surveillance to Identify California Alternative and Emerging Tobacco Industry Policy Influence and Mobilization on Facebook
Source: Int J Environ Res Public Health. 2021 Oct 23;18(21):11150. doi: 10.3390/ijerph182111150 (PMC8583030; doi:10.3390/ijerph182111150)
Supplement: Supplementary file 1 [file ijerph-18-11150-s001.zip › ijerph-1399981-supplementary.pdf]

Supplementary File

Figure S1: Facebook posts engagements typologies.

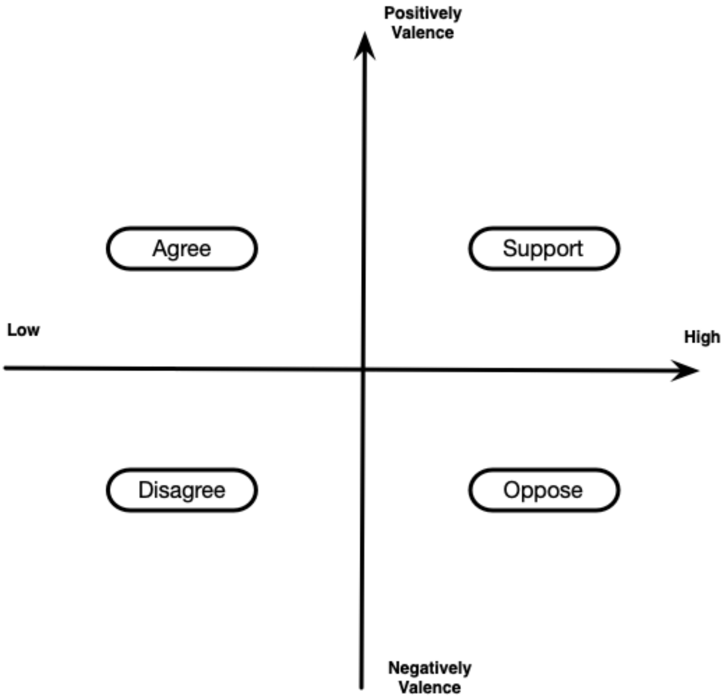

Description: The vertical axes represent the attitude toward to posts which opposes the tobacco control policy, and the abscissa axis represent the active level of the engagement. positive engagement levels are high active (Support) and low active (Agree) and negative engagement levels are also high active (Oppose) and low active (Disagree) resulting in classification of different engagement behavior that varies in intensity and valence

Table S1. Inductive code list

CODE	SUBCODE	DESCRIPTION
**Call to political action**	advocate	Call to action is general advocacy
	appear	Call to action to appear at some event (policy meeting, rally, etc.)
	call	Call to action to make a phone call
	donate money	Call to action to donate money to an organization
	face to face with policymakers	Call to action for face to face contact with policymakers (e.g., lobbying, legislative visit, etc.). Does not include appearing at meetings.
	inform non-policy makers	Call to action to inform, talk to, dialogue with non-policy makers (e.g., friends, family) about vaping. Purpose is to sway public opinion to increase acceptability of vaping.
	join organization	Call to action to join the posting organization (CCASAA or NC-SFATA or parent organizations) or other related organizations
	lawsuit (as party or supporter)	Call to action related to legal intervention
	moderate Facebook group	Call to action to moderate the Facebook group
	public comment	Call to action to make a public comment about vaping (setting is not specified). Comment can be in writing or verbal.
	purchase merchandise	Call to action to purchase merchandise of some type.
	share testimonial	Call to action to share testimonial as a personal experience related to vaping or as a retailer. Sharing can be in policy arena, to others interpersonally, or publicly in non-policy setting
	subscribe_newsletter	Call to action to subscribe to a newsletter
	survey participation	Call to action to participate in a survey
	vote	Call to action to vote
	website blackout	Call to action to alter (retail) website to be blacked out
	write	Call to action to write (email, letter, web post, other) to a policymaker or other decision making audience.
**SHARE: Action taken**	action taken_call	Describes advocacy call made by an individual

	action taken_testify	Describes advocacy testimony made by an individual
	action taken_write	Describes written advocacy action taken by an individual
**SHARE: Advocacy experience**	experience with advocacy_negative	Negative experience an individual has in the process of engaging in advocacy
	experiences with advocacy	Experience an individual has in the process of engaging in advocacy
**SHARE: Event**	industry event	E-cigarette industry event not related to the parent organization (e.g., CASAA or SFATA)
	invitation_advocacy_federal	Invitation to participate in a federal advocacy event.
	invitation_advocacy_state	Invitation to participate in a state advocacy event.
	invitation_sfata meeting	Invitation to attend a SFATA chapter meeting or SFATA-sponsored event
**SHARE: Misc**	anti-vaping material	Anti-vaping material (print or video).
	consumer information	Information for consumers related to vaping behavior and not about policy issues
	information_CA political districts	Information on California political districts
	presentation on vaping	Information about a presentation about vaping being given.
	presidential candidate position on ecigs	Information on Democratic nominees for president summarizing views on vaping regulation
	response from policymaker	Response from a policymaker regarding advocacy effort on vaping issue
	running for president	Facebook group member campaigning for President
	vape paraphernalia	Vape paraphernalia
**SHARE: News**	news_adverse outcomes	News about adverse outcomes related to vaping
	news_increase in vaping	News about increases in vaping
	news_vape shops	News related to vape shops
	news_youth vaping	News related to youth vaping
	newsletter	Organizational newsletter (or link to a newsletter).
**SHARE: Opinion**	opinion_others smoking behavior	Opinion expressed about the smoking behavior of another person
	opinion_policies	Opinion expressed about vaping related policy/policies

**SHARE: Organizational action**		Documentation of organizational activity (CCASAA or NC-SFATA) (e.g., hosting events, paid media articles, public testimony, giving interviews, lobbying, etc.) to advance the organization's mission.
**SHARE: Other groups**	organizational websites	Information or links to the organizational website
	other pro-vape organization	Information about or links to other pro-vape organizations
	tobacco control group actions	Actions of tobacco control advocates
**SHARE: Policy update**	policy update	Policy update, but the level of government not specified.
	policy update_federal	Policy update at the federal level.
	policy update_local	Policy update at the local level.
	policy update_retail	Policy update related to retailers
	policy update_state	Policy update at the state level.
**SHARE: Retail**	retailer information	Information for retailers for conduct of business (e.g., how to be in compliance with regulatory or tax laws).
	retailer practices	Other retailer’s business practices.
**SHARE: Science**		Science of vaping (including medical information, epidemiological information, interpretation of data of study findings, results of studies, or other discussions about "evidence" for and against vaping).

Table S2. Local ordinances and state and federal legislation discussed in Facebook groups.

Organization	Local Ordinances	California	Federal
CCASSA	Cities:<br><br>Adelanto<br>Albany<br>Alturas<br>Anderson<br>Arroyo Grande<br>Auburn<br>Belmont Beverly Hills<br>Burbank<br>Burlingame, Capitola<br>Carpinteria<br>Cerritos<br>Chico<br>Chino<br>Chula Vista<br>Concord<br>Coronado<br>Culver City<br>Cupertino<br>Danville<br>Davis<br>El Cajon<br>El Monte<br>Fremont<br>Grand Terrace<br>Hawaiian Gardens<br>Hermosa Beach<br>Imperial Beach<br>Irvine<br>Lafayette<br>Laguna Niguel<br>Larkspur Ontario<br>Livermore<br>Long Beach<br>Loomis<br>Los Angeles<br>Los Gatos, Mammoth Lakes<br>Manhattan Beach<br>Menlo Park<br>Mill Valley<br>Morgan Hill<br>Morro Bay<br>Oakland<br>Oroville<br>Oxnard<br>Pacifica<br>Pacific Grove<br>Palmdale	A.B. 131 (2017-18)<br>A.B. 1639 (2019-20)<br>S.B. 38 (2019-20)<br>S.B. 39 (2019-20)<br>S.B. 538 (2019-20)<br>S.B. 793 (2019-20)	H.R. 293 (2019-20)<br>H.R. 2411 (2019-20)<br>FDA Tobacco Product Standard for Nicotine Level of Combusted Cigarettes propose rulemaking<br>Objection to OMB regarding CDC "Tips from Smokers" campaign

	Palo Alto<br>Pasadena<br>Pismo Beach<br>Pittsburg<br>Pleasanton<br>Pomona<br>Rancho Mirage<br>Redondo Beach<br>Redwood City<br>Richmond<br>Sacramento<br>Saint Helena<br>San Anselmo<br>San Carlos<br>San Clemente<br>San Jose<br>San Juan Capistrano<br>San Luis Obispo<br>San Marcos<br>San Mateo<br>San Rafael<br>Santa Maria<br>Santa Monica<br>Seaside<br>Solana Beach<br>Solvang<br>Sunnyvale<br>Upland<br>Torrance<br>Tracy<br>Ventura<br>Visalia<br>Watsonville<br>West Hollywood<br>West Sacramento<br>Woodland<br><br>Counties:<br><br>Alameda County<br>Butte County<br>Contra Costa County<br>Lake County<br>Los Angeles County<br>Marin County<br>Sacramento County<br>San Francisco County<br>San Diego County San<br>Luis Obispo County<br>San Mateo County<br>Santa Barbara County<br>Santa Clara County<br>Santa Cruz County		

	Shasta County<br>Sonoma County<br>Ventura County		
NC-SFATA	Sonoma<br>Turlock<br>Yountville<br><br><br>Alameda County<br>Contra Costa County<br>San Mateo County<br>Shasta County	A.B.X. 26 (2015-16)<br>A.B. 768 (2015-16)<br>A.B. 1594 (2015-16)<br>S.B. 24 (2015-16)<br>S.B.X. 25 (2015-16)<br>S.B. 140 (2015-16)<br>S.B. 151 (2015-16)<br>S.B. 1333 (2015-16)<br>Proposition 56 (2016)	H.R. 2058 (2015-16)<br>H.R. 1136 (2015-16)

Table S3: Examples of industry interference Facebook post themes

Political interference type	Code	Subcode	CCASAA (number of posts)	NCSFATA (number of posts)
Information	Call to political action	advocate	5	0
		appear	40	11
		call	16	7
		face to face with policymakers	1	5
		inform non-policy makers	1	
		public comment	7	2
		share testimonial	8	0
		vote	2	6
		website blackout	0	1
		write	47	6
	SHARE: Event	invitation_advocacy_federal	0	3
		invitation_advocacy_state	0	3
		invitation_sfata meeting	0	36
	SHARE: Science		38	14
	SHARE: Action taken	action taken_call	1	0
		action taken_testify	0	1
		action taken_write	1	0
	SHARE: Advocacy experience	experience with advocacy_negative	2	0
		experiences with advocacy	3	5
Policy substitution	SHARE: Opinion	opinion_policies	34	17
Constituency building	SHARE: Other groups	organizational websites	1	1
		other pro-vape organization	3	2
	SHARE: Policy update	policy update	4	1
		policy update_federal	13	12
		policy update_local	233	22
		policy update_retail	1	0
		policy update_state	56	25
	Call to action	join organization	38	2
Legal	Call to action	lawsuit (as party or supporter)	3	1
